# Development of an Advanced Synthetic Route to Macrosphelides and Its Application to the Discovery of a More Potent Macrosphelide Derivative

**DOI:** 10.3390/molecules191015572

**Published:** 2014-09-29

**Authors:** Yu Mi Heo, Hunseok Lee, Young Kee Shin, Seung-Mann Paek

**Affiliations:** 1 College of Pharmacy and Research Institute of Pharmaceutical Sciences, Gyeongsang National University, Jinju daero, Jinju 660-701, Gyeongnam, Korea; E-Mail: ymh@gnu.ac.kr; 2 College of Pharmacy, Seoul National University, Seoul 151-742, Korea; E-Mails: ryanlee0@snu.ac.kr (H.L.); ykeeshin@snu.ac.kr (Y.K.S.)

**Keywords:** macrosphelides, natural product, total synthesis

## Abstract

The discovery of a more cytotoxic macrosphelide derivative, including its total synthesis and bioassay are described. Application of the Koide protocol to a readily available propagylic alcohol allowed the rapid and practical synthesis of a macrosphelide A skeleton. This strategy enabled the successful improvement of the cytotoxic activity of the macrosphelide derivative.

## 1. Introduction

Natural products have been tapped as an endless supply of resources for new drug development, because of their potency and safety [1,2]. Actually, over the past three decades, approximately 50% of new drugs have been developed from natural products [3,4]. However, some natural products with low potency or high toxicity still need derivatization based on their structure-activity relationships (SAR) and chemical modifications [5].

**Figure 1:**
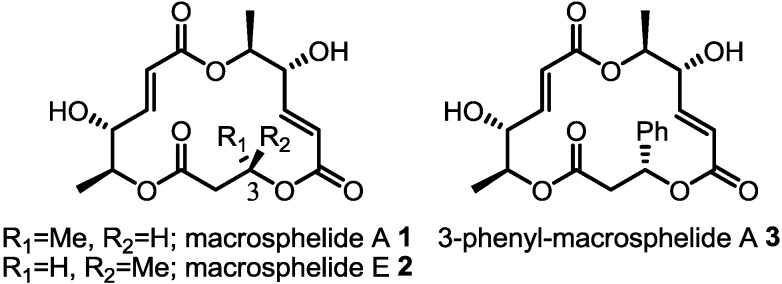
Structures of macrosphelide A, E and 3-phenylmacrosphelide A.

Since its first isolation in 1995, macrosphelide A (**1**) has been expected to serve as a new lead compound because of its safety, anticancer activity and unique chemical skeleton [6,7,8,9]. However, its low potency still hampers further developments. To solve this limitation, a variety of approaches including total synthesis [10,11,12,13,14,15,16,17,18,19,20], medicinal chemistry [21,22,23], combinatorial chemistry [24], and fluorinated chemistry [25] have been carried out to establish the SAR and discover more promising derivatives of **1**. As a result, it has been possible to improve its various biological activities slightly after extensive efforts, including the confirmation of natural isomer structures and the discovery of potent derivatives such as aza-**1**, ring size modified-**1**, fluorinated-**1** and thiazoline linked-**1**. However, it is noteworthy that C3-modified derivatives of **1** without changing the ring skeleton have not been studied extensively [21,26]. Considering the similar potency of macrosphelide A (**1**) and E (**2**), it can be hypothesized that the C3 substituent can be altered without loss of activity (Figure 1) [27,28]. Moreover, modification of this carbon can be carried out to improve lipophilicity and cell permeability or suppress metabolism, such as by esterase mediated hydrolysis. With this possibility for chemical modification in mind, we sought to modify the C3 position of **1**, because this SAR data can be combined with other SAR information to develop more potent derivatives. For the purpose of this research, a practical synthetic route to the macrosphelide skeleton had to be established. Herein, we would like to report the discovery of 3-phenyl substituted macrosphelide A (**3**), which is much more potent than macrosphelide A **1**.

## 2. Results and Discussion

### 2.1. Retrosynthesis

**Scheme 1:**
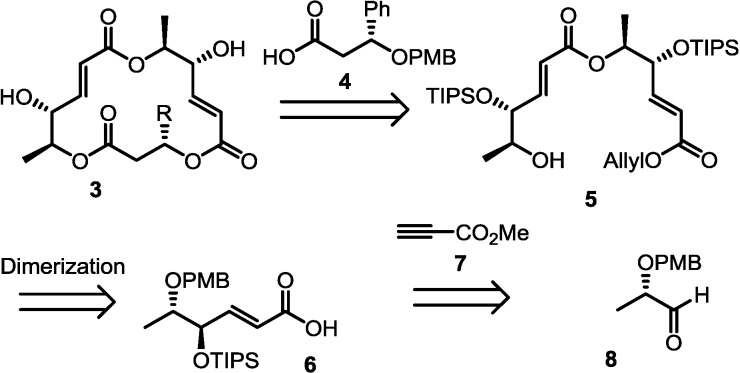
Retrosynthesis to 3-phenyl-macrosphelides **3**.

The retrosynthesis is outlined in Scheme 1. Considering the convergent synthesis of **3**, it was envisioned that esterification of dimeric alcohol **5** with carboxylic acid **4** would produce a unique macrosphelide skeleton efficiently.

Dimeric alcohol **5** was expected to be transformed via a simple deprotection/esterification sequence of bis-protected unsaturated acid **6**. Finally, employment of the Koide protocol [29] to the PMB-protected aldehyde **8** was designed to yield the key monomeric acid **6** because this strategy can introduce a three-carbon unit directly using commercially available resources.

### 2.2. Synthesis of Monomer **6** of Macrosphelide A

**Scheme 2:**
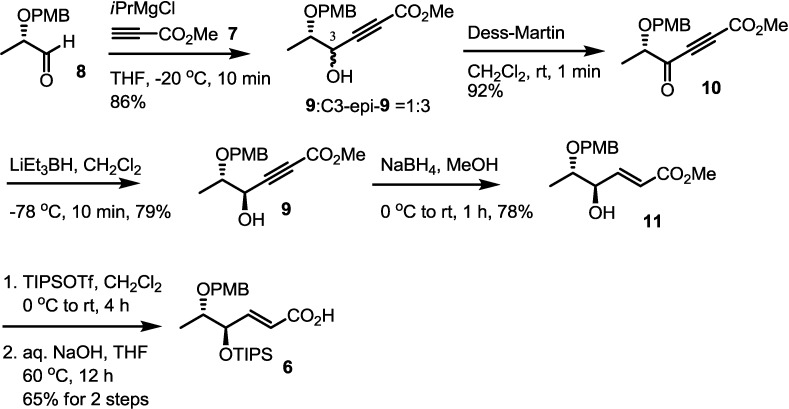
Synthesis of monomer **6**.

Synthesis of monomer **6** commenced with the three-carbon homologation of the PMB-protected aldehyde **8** by reaction of **7** [30] (Scheme 2). Because use of normal bases such as LiHMDS or NaHMDS produced the desired addition product **9** in low yield, exploration of conditions for this conversion was carried out. After an intensive screening of bases and solvents, *i*PrMgCl was found to produce the corresponding propargylic alcohol **9**. Although the desired diastereomer **9** was produced as a minor isomer, this facial selectivity problem could be solved via an oxidation/stereoselective reduction sequence. Dess-Martin oxidation of the diastereomeric mixture **9**/C3-epi-**9** produced alkynone **10** in good yield. With alkynone **10** in hand, stereoselective reduction of the ketone moiety was executed. Based on the reaction pattern of similar systems [17,31], Super-Hydride was chosen to obtain a chelation-controlled reduction product. Gratifyingly, the desired secondary alcohol **9** was obtained exclusively as the desired isomer, which was subjected to the Koide procedure.

When the propargylic alcohol **9** in methanol was treated with NaBH_4_, the desired *trans*-allylic alcohol **11** could be obtained in excellent yield, as was previously reported for a similar skeleton [32]. Moreover, this reaction could be performed at a more convenient temperature without loss of chemical yield. Considering the mildness of the reducing agent and ease of the reaction handling, this stereoselective reduction sequence is ideal for the efficient synthesis of macrosphelides and their derivatives. Finally, TIPS protection followed by hydrolysis of the allylic alcohol **11** produced **6** in excellent yield. Employing this synthetic route, the key building block **6** could be prepared on multi-gram scale.

### 2.3. Completion of the Synthesis

**Scheme 3:**
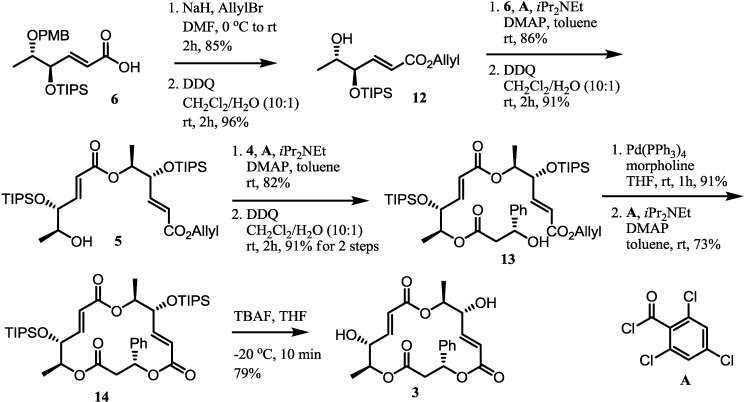
Total synthesis of 3-phenylmacrosphelide A (**3**).

Completion of synthesis was performed by the iterative ligation of three hydroxy acid fragments (Scheme 3). *O*-Allylation and Yonemitsu PMB deprotection [33] of the bis-protected carboxylic acid **6** produced hydroxyester **12**, which was transformed into diester **5** by Yamaguchi esterification and PMB deprotection. Stepwise esterification of the known carboxylic acid **4** [34] with the alcohol **5**, followed by PMB deprotection produced trimeric alcohol **13** in excellent yield. Pd-catalyzed allyl deprotection and Yamaguchi lactonization produced the bis-TIPS protected 3-phenylmacrosphelide A **14**. Finally, treatment of **14** with TBAF at a low temperature produced the target molecule **3**.

### 2.4. Cytotoxic Activity

**Figure 2:**
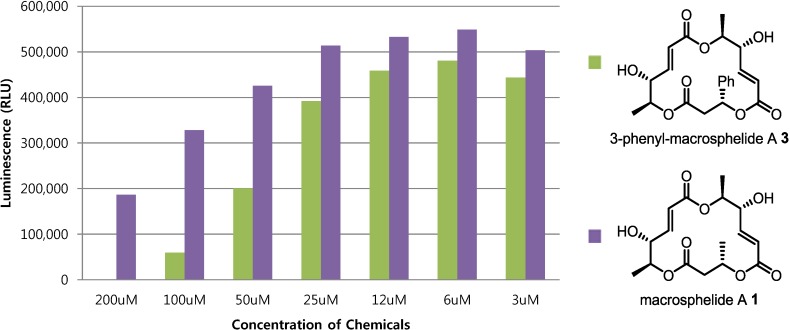
Anticancer activity of **1** and **3**.

The cytotoxic activities of **1** and **3** are shown in Figure 2. Cell viability was determined with the use of a luminescent cell viability assay kit. After 4 × 10^3^ SKOV3 cells were seeded in an opaque-walled 96-well microplate, the cells were treated with **1** and **3** and incubated at 37 °C for 72 h. For equilibration of the microplate, it was incubated for 30 min at room temperature. Thereafter, 100 µL CellTiter-Glo^®^ reagent was added to 100 µL of medium containing cells and the plate was mixed for 2 min on an orbital shaker to induce cell lysis. Finally, the plate was incubated for 10 min at room temperature and the luminescence was recorded by a GENios reader. From this assay, a remarkable improvement in cytotoxic activity could be observed. When cells were treated with **3** instead of **1**, cytotoxicity in the carcinoma cell line was increased (see Figure 2). This result means that C3 modification of the macrosphelide skeleton could contribute to an improvement of cytotoxic activity, which is strongly required for macrosphelide-related anticancer drug development.

## 3. Experimental Section

### 3.1. General Information

Unless noted otherwise, all starting materials and reagents were obtained from commercial suppliers and were used without further purification. Tetrahydrofuran and Et_2_O were distilled from sodium benzophenone ketyl. Dichloromethane was freshly distilled from calcium hydride. All solvents used for routine isolation of products and chromatography were reagent grade and glass distilled. Air and moisture sensitive reactions were performed under an argon atmosphere. Flash column chromatography was performed using silica gel 60 (230–400 mesh, Merck, Darmstadt, Germany) with the indicated solvents. Thin*-*layer chromatography was performed using 0.25 mm silica gel plates (Merck). ^1^H-NMR data were reported in the order of chemical shift, multiplicity (s, singlet; d, doublet; t, triplet; q, quartet; m, multiplet and/or multiple resonance), number of protons, and coupling constant in hertz (Hz).

*(5S)-Methyl 4-hydroxy-5-(4-methoxybenzyloxy)hex-2-ynoate* (**9**). To a solution of methyl propiolate (540 mg, 6.4 mmol) in THF (10 mL), iPrMgCl (2.0 M in THF, 3.2 mL, 6.4 mmol) was added at −20 °C. After stirring at the same temperature for 30 min, a solution of aldehyde **8** (340 mg, 1.8 mmol) in THF (5 mL) was added at −20 °C. The reaction mixture was stirred for 1 h at the same temperature and quenched with aq. NH_4_Cl. The reaction mixture was extracted with EtOAc and the combined organic layers were dried over MgSO_4_, filtered and concentrated *in vacuo*. The residue was purified by flash column chromatography on silica gel (EtOAc/*n*-hexane = 1:5 to 1:2) to afford 420 mg (86%) of propargylic alcohol **9** as inseparable diastereomers: FT-IR (KBr) ν_max_ 3424, 2953, 2236, 1715, 1512 cm^−1^; ^1^H-NMR (CDCl_3_, 500 MHz, 1:1 mixture of diasteromers) δ 7.30 (d, 2H, *J* = 8.5 Hz), 6.90 (d, 2H, *J* = 8.5 Hz), 4.63 (m, 1H), 4.50 (m, 1H), 3.83 (s, 3H), 3.80 (s, 3H), 3.48 (m, 2H), 3.73 (m, 1H), 1.37 (d, 3H, *J* = 6.5 Hz); LR-MS (ESI+) *m/z* 317 (M+K^+^).

*(4R,5S,E)-Methyl 4-hydroxy-5-(4-methoxybenzyloxy)hex-2-enoate* (**11**). To a solution of propargylic alcohol **9** (415 mg, 1.5 mmol, mixture of diastereomers) in CH_2_Cl_2_ (10 mL), Dess-Martin periodinane (760 mg, 1.8 mmol) was added at room temperature. After stirring for 10 min, the reaction mixture was quenched with saturated aq. NaHCO_3_, and then extracted with CH_2_Cl_2_. The combined organic layers were dried over MgSO_4_, filtered and concentrated *in vacuo*. The residue was purified by flash column chromatography on silica gel (EtOAc/*n*-hexane = 1:5 to 1:2) to afford 380 mg (92%) of slightly unstable ketone **10** as a yellow oil: ^1^H-NMR (CDCl_3_, 500 MHz) δ 7.21 (d, 2H, *J* = 8.5 Hz), 6.81 (d, 2H, *J* = 8.5 Hz), 4.55 (d, 1H, *J* = 10.5 Hz), 4.36 (dt, 2H, *J* = 10.5 Hz), 3.77 (s, 3H), 3.72 (s, 3H), 1.34 (d, 3H, *J* = 6.5 Hz). To a solution of ketone **10** (280 mg, 1.0 mmol) in CH_2_Cl_2_ (10 mL), Super-Hydride (1.0 M in THF, 1.1 mL, 1.1 mmol) was added slowly at −78 °C. After stirring for 5 min, the reaction mixture was quenched with saturated aqueous NH_4_Cl, and then extracted with CH_2_Cl_2_. The combined organic layers were dried over MgSO_4_, filtered and concentrated *in vacuo*. The residue was purified by flash column chromatography on silica gel (EtOAc/*n*-hexane = 1:2) to afford 260 mg (90%) of propargylic alcohol **9** as a colorless oil. A diastereoselectivity (>10:1) was confirmed by proton NMR analysis.

To a solution of propargylic alcohol **9** (210 mg, 0.76 mmol) in MeOH (3 mL), NaBH_4_ (29 mg, 0.76 mmol) was added at 0 °C. After stirring for 2 h, the reaction mixture was quenched with saturated aq. NH_4_Cl, and then extracted with EtOAc. The combined organic layers were dried over MgSO_4_, filtered and concentrated *in vacuo*. The residue was purified by flash column chromatography on silica gel (EtOAc/*n*-hexane = 1:2) to afford 160 mg (75%) of allylic alcohol **11** as a colorless oil; 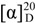 −18.9 (c 0.76, CHCl_3_); FT-IR (KBr) ν_max_ 2949, 1771, 1720, 1610 cm−1; ^1^H-NMR (CDCl_3_, 300 MHz) δ 7.26 (d, 2H, *J* = 6.3 Hz), 6.93–6.88 (m, 3H), 6.15 (dd, 1H, *J* = 15.6, 1.8 Hz), 4.65–4.44 (m, 3H), 3.83 (s, 3H), 3.76 (s, 3H), 3.70 (m, 1H), 2.37 (d, 1H, *J* = 4.5 Hz), 1.15 (d, 3H, *J* = 6.5 Hz); ^13^C-NMR (CDCl_3_, 75 MHz) δ 166.7, 159.3, 145.9, 129.9, 129.3, 121.4, 113.9, 76.2, 72.6, 70.5, 55.2, 51.5, 14.0.

*(4R,5S,E)-5-(4-Methoxybenzyloxy)-4-(triisopropylsilyloxy)hex-2-enoic acid* (**6**). To a solution of allylic alcohol **11** (15 mg, 55 μmol) in CH_2_Cl_2_ (1 mL), *i*Pr_2_NEt (14 mg, 110 μmol) and TIPSOTf (20 mg, 66 μmol) were added at 0 °C. After stirring for 5 h, the reaction mixture was quenched with aq. NH_4_Cl, and then extracted with EtOAc. The combined organic layers were dried over MgSO_4_, filtered and concentrated *in vacuo*. The residue was used in the next step without further purification.

A solution of the crude mixture from the previous reaction in THF/H_2_O (3 mL/1 mL) was treated with NaOH (10% in H_2_O, 0.2 mL) and warmed to 50 °C for 12 h. The reaction mixture was quenched with 1N HCl and then extracted with EtOAc (3 times). The combined organic layers were dried over MgSO_4_, filtered and concentrated *in vacuo*. The residue was purified by flash column chromatography on silica gel (EtOAc/*n*-hexane = 1:1 to EtOAc only) to afford 15 mg (65% for 2 steps) of carboxylic acid **6** as a colorless oil. 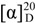 −9.0 (c 1.0, CHCl_3_); FT-IR (KBr) ν_max_ 2942, 2865, 2678, 1698, 1655 cm^−1^; ^1^H-NMR (CDCl_3_, 500 MHz) δ 7.26 (d, 2H, *J* = 6.5 Hz), 7.11 (dd, 1H, *J* = 15.5, 5.5 Hz), 6.88 (d, 2H, *J* = 6.5 Hz), 6.08 (dd, 1H, *J* = 16.0, 1.0 Hz), 4.55 (d, 2H, *J* = 4.0 Hz), 4.47 (m, 1H), 3.82 (s, 3H), 3.60 (m, 1H), 1.28 (d, 3H, *J* = 6.6 Hz), 1.17 (m, 21H); ^13^C-NMR (CDCl_3_, 125 MHz) δ 159.5, 151.5, 130.9, 129.6, 114.2, 114.1, 78.4, 76.3, 71.7, 55.6, 18.4 (2C), 16.1, 13.1; LR-MS (ESI) *m/z* 461 (M+K^+^).

*(4R,5S,E)-Allyl 5-hydroxy-4-(triisopropylsilyloxy)hex-2-enoate* (**12**). To a solution of carboxylic acid **6** (480 mg, 1.1 mmol) in DMF (5 mL), Cs_2_CO_3_ (740 mg, 2.3 mmol) and allyl bromide (0.15 mL, 1.7 mmol) were added at 0 °C. After stirring for 10 min at the same temperature, the reaction mixture was quenched with saturated aq. NH_4_Cl. The reaction mixture was diluted with EtOAc and washed with H_2_O (3 times) and the organic layers was dried over MgSO_4_, filtered and concentrated *in vacuo*. The residue was purified by flash column chromatography on silica gel (EtOAc/*n*-hexane = 1:5, R_f_ 0.8) to afford 450 mg of allyl with small amount of impurities. To a solution of this ester in CH_2_Cl_2_/H_2_O (20 mL/1 mL), DDQ (260 mg, 1.2 mmol) was added at ambient temperature. After stirring for 30 min, the reaction mixture was quenched with aq. NaHCO_3_, and extracted with CH_2_Cl_2_. The organic layers were dried over MgSO_4_, filtered and concentrated *in vacuo*. The residue was purified by flash column chromatography on silica gel (EtOAc/*n*-hexane = 1:10 to 1:5) to afford 320 mg (81% for 2 steps) of monomeric alcohol **12** as a colorless oil: 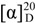 −12.3 (c 0.8, CHCl_3_); FT-IR (KBr) ν_max_ 2942, 2866, 1360, 1819, 1721 cm^−1^; ^1^H-NMR (CDCl_3_, 300 MHz) δ 6.96 (dd, 1H, *J* = 15.6, 6.3 Hz), 6.04 (dd, 1H, *J* = 15.6, 1.2 Hz), 5.92 (m, 1H), 5.30 (m, 2H), 4.66 (d, 2H, *J* = 5.7 Hz), 4.35 (m, 1H), 3.92 (m, 1H), 1.13 (d, 3H, *J* = 6.6 Hz); ^13^C-NMR (CDCl_3_, 75 MHz) δ 165.5, 146.4, 132.1, 122.6, 118.1, 76.1, 70.7, 65.1, 17.9, 17.4, 12.3; LR-MS (FAB+) *m/z* 343 (M+H^+^).

*(4R,5S,E)-Allyl 5-((4R,5S,E)-5-hydroxy-4-(triisopropylsilyloxy)hex-2-enoyloxy)-4-(triisopropylsilyloxy)hex-2-enoate* (**5**). To a solution of carboxylic acid **6** (410 mg, 0.97 mmol) in toluene (5 mL), *i*Pr_2_NEt (0.34 mL, 1.9 mmol) and 2,4,6-trichlorobenzoyl chloride (0.18 mL, 1.2 mmol) were added at 0 °C. After stirring for 1 h, DMAP (240 mg, 1.9 mmol) was added at ambient temperature. After stirring for 10 min, a solution of monomeric alcohol **12** (320 mg, 0.97 mmol) in toluene (5 mL) was added and stirred for 1 h. The reaction mixture was quenched with aq. NH_4_Cl and then extracted with EtOAc (3 times). The combined organic layers were dried over MgSO_4_, filtered and concentrated *in vacuo*. The residue was filtered by flash column chromatography on silica gel (EtOAc/*n*-hexane = 1:5, R_f_ 0.7) to afford 610 mg (84%) of dimeric ester as a colorless oil.

To a solution of dimeric ester (610 mg, 81 mmol) in CH_2_Cl_2_/H_2_O (30 mL/2 mL), DDQ (260 mg, 1.2 mmol) was added at ambient temperature. After stirring for 30 min and the reaction mixture was quenched with aq. NaHCO_3_, and extracted with CH_2_Cl_2_. The organic layers were dried over MgSO_4_, filtered and concentrated *in vacuo*. The residue was purified by flash column chromatography on silica gel (EtOAc/*n*-hexane = 1:5) to afford 493 mg (97%) of dimeric alcohol **13** as a colorless oil: 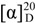 −14.2 (c 0.76, CHCl_3_); FT-IR (KBr) ν_max_ 2942, 2866, 1359, 1723, 1655 cm^−1^; ^1^H-NMR (CDCl_3_, 500 MHz) δ 6.89 (m, 2H), 6.00 (dd, 1H, *J* = 15.3, 1.2 Hz), 5.96 (dd, 1H, *J* = 15.3, 1.2 Hz), 5.88 (m, 1H), 5.24 (m, 1H), 5.00 (dd, 1H, *J* = 8.5, 6.5 Hz), 4.58 (d, 2H, *J* = 3.3 Hz), 4.52 (m, 1H), 4.28 (m, 1H), 3.84 (dd, 1H, *J* = 6.5, 3.5 Hz), 1.16 (d, 3H, *J* = 6.6 Hz), 1.04 (d, 3H, *J* = 6.4 Hz), 0.98 (m, 21H); ^13^C-NMR (CDCl_3_, 125 MHz) δ 166.0, 165.8, 147.5, 146.9, 132.5, 123.1, 122.6, 118.4, 76.4, 74.6, 73.6, 71.1, 65.5, 60.7, 18.3, 17.8, 14.5, 12.8, 12.7; LR-MS (ESI) *m/z* 649 (M+Na^+^).

*(4R,5S,E)-Allyl 5-((4R,5S,E)-5-((R)-3-hydroxy-3-phenylpropanoyloxy)-4-(triisopropylsilyloxy)hex-2-enoyloxy)-4-(triisopropylsilyloxy)hex-2-enoate* (**13**). To a solution of carboxylic acid **5** (130 mg, 0.45 mmol) in toluene (3 mL), *i*Pr_2_NEt (0.13 mL, 0.75 mmol) and 2,4,6-trichlorobenzoyl chloride (0.070 mL, 0.45 mmol) were added at 0 °C. After stirring for 1 h, DMAP (91 mg, 0.75 mmol) was added at ambient temperature. After stirring for 10 min, a solution of dimeric alcohol **13** (240 mg, 0.38 mmol) in toluene (2 mL) was added and stirred for 1 h. The reaction mixture was quenched with aq. NH_4_Cl and then extracted with EtOAc. The combined organic layers were dried over MgSO_4_, filtered and concentrated *in vacuo*. The residue was purified by flash column chromatography on silica gel (EtOAc/*n*-hexane = 1:1) to afford 278 mg (82%) of trimeric ester as a colorless oil. To a solution of trimeric ester (75 mg, 84 mmol) in CH_2_Cl_2_/H_2_O (3 mL/0.3 mL), DDQ (21 mg, 0.092 mmol) was added at ambient temperature. After stirring for 30 min and the reaction mixture was quenched with aq. NaHCO_3_ and extracted with CH_2_Cl_2_ (3 times). The combined organic layers were dried over MgSO_4_, filtered and concentrated *in vacuo*. The residue was purified by flash column chromatography on silica gel (EtOAc/*n*-hexane = 1:10 to 1:5) to afford 72 mg of trimeric alcohol **16**; 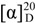 −0.5 (c 0.86, CHCl_3_); ^1^H-NMR (CDCl_3_, 500 MHz) δ 7.36 (m, 5H), 6.97 (dd, 1H, *J* = 15.5, 5.5 Hz), 6.92 (dd, 1H, *J* = 15.5, 5.5 Hz), 6.11 (m, 2H), 5.95 (m, 1H), 5.23 (m, 2H), 5.15 (dd, 1H, *J* = 8.0, 4.5 Hz), 5.00 (m, 2H), 4.68 (m, 2H), 4.63 (m, 1H), 4.58 (m, 1H), 2.75 (m, 2H), 1.45 (d, 3H, *J* = 6.6 Hz), 1.35 (d, 3H, *J* = 6.5 Hz), 1.07 (m, 42H); LR-MS (FAB+) *m/z* 775 (M+H^+^).

*(4R,7E,9R,10S,13E,15R,16S)-10,16-Dimethyl-4-phenyl-9,15-bis(triisopropylsilyloxy)-1,5,11-trioxa-cyclohexadeca-7,13-diene-2,6,12-trione* (**14**). To a solution of hydroxyl allyl ester **14** (72 mg) in THF (3 mL) were added Pd(PPh_3_)_4_ (30 mg, 0.028 mmol) and morpholine (0.10 mL, excess) at ambient temperature. The mixture was stirred for 10 min at the same temperature, quenched with 1 N HCl and extracted with EtOAc (3 times). The organic layers were dried over MgSO_4_, filtered and concentrated in vacuo. The residue was purified by flash column chromatography on silica gel (EtOAc/*n*-hexane = 1:2 to EtOAc only) to afford 62 mg of crude seco acid as a colorless oil. To a solution of crude acid (62 mg) in toluene (2 mL) were added *i*Pr_2_NEt (94 μL, 0.54 mmol) and 2,4,6-trichlorobenzoyl chloride (40 μL, 0.27 mmol) at room temperature and the reaction was stirred for 2 h. To this reaction mixture, DMAP (66 mg, 0.54 mmol) was added and warmed to 80 °C. After stirring for 12 h, the mixture was quenched with aq. NH_4_Cl and extracted with EtOAc (3 times). The combined organic layers were dried over MgSO_4_ and concentrated *in vacuo*. The residue was purified by flash column chromatography on silica gel (EtOAc/*n*-hexane = 1:10) to afford 35 mg (57% for 4 steps) of bis-TIPS protected phenyl macrosphelide **17** as a pale yellow oil.; 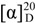 −6.7 (c 0.76, CHCl_3_); FT-IR (KBr) ν_max_ 2942, 2866, 2359, 1721 cm−1; ^1^H-NMR (CDCl_3_, 500 MHz) δ 7.36 (m, 5H), 6.88 (m, 2H), 6.22 (dd, 1H, *J* = 8.5, 2.5 Hz), 5.96 (d, 1H, *J* = 15.5 Hz), 5.93 (d, 1H, *J* = 15.5 Hz), 5.02 (m, 1H), 4.91 (m, 1H), 4.28 (t, 1H, *J* = 7.5 Hz), 4.24 (t, 1H, *J* = 7.5 Hz), 2.89 (dd, 1H, *J* = 15.5, 10.0 Hz), 2.75 (dd, 1H, *J* = 15.5, 2.5 Hz), 1.45 (d, 3H, *J* = 6.6 Hz), 1.35 (d, 3H, *J* = 6.5 Hz), 1.07 (m, 42H); ^13^C-NMR (CDCl_3_, 125 MHz) δ 169.5, 164.9, 164.3, 148.5, 147.9, 139.5, 129.0, 128.6, 126.4, 123.0, 122.6, 76.4, 74.9, 74.0, 73.0, 72.4, 42.3, 31.9, 18.3, 18.2, 18.1, 12.9, 12.8; LR-MS (FAB+) *m/z* 717 (M+H^+^).

*3-Phenylmacrosphelide A* (**3**). To a solution of bis-TIPS protected macrosphelide **17** (18 mg, 25 μmol) in THF (1 mL), TBAF (1.0 M in THF, 75 μL, 75 μmol) was added at −20 °C. After stirring for 10 min, the mixture was quenched with aq. NH_4_Cl and extracted with EtOAc. The organic layers were dried over MgSO_4_, filtered and concentrated *in vacuo*. The residue was purified by flash column chromatography on silica gel (EtOAc/*n*-hexane = 1:1) to afford 8.0 mg (79%) of phenyl macrosphelide 3 as a white solid: 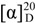 +34.2 (c 0.21, CHCl_3_); ^1^H-NMR (CDCl_3_, 500 MHz) δ 7.39–7.31 (m, 5H), 7.02 (dd, 1H, *J* = 16.0, 4.0 Hz), 6.95 (dd, 1H, *J* = 16.0, 4.0 Hz), 6.31 (dd, 1H, *J* = 11.5, 2.0 Hz), 6.12 (dd, 1H, *J* = 16.0, 1.5 Hz ), 6.10 (dd, 1H, *J* = 16.0, 1.5 Hz ), 5.04–4.96 (m, 1H), 4.94–4.89 (m, 1H), 4.28 (m, 1H), 4.18 (m, 1H), 3.03 (dd, 1H, *J* = 16.0, 11.5 Hz ), 2.94 (br, 1H), 2.75 (dd, 1H, *J* = 16.0, 2.0 Hz ), 2.50 (br, 1H), 1.50 (d, 3H, *J* = 6.5 Hz), 1.41 (d, 3H, *J* = 6.5 Hz); ^13^C-NMR (CDCl_3_, 125 MHz) δ170.1, 166.3, 164.3, 146.6, 145.5, 139.3, 129.1, 128.8, 126.5, 122.7 (2C), 75.9, 75.1, 74.5, 73.6, 72.5, 42.0, 18.5, 18.4; FT-IR (KBr) ν_max_ 3429, 2923, 2852, 1710, 1627; LR-MS (FAB) *m/z* 405 (M+H^+^); HR-MS (FAB) calcd for C_21_H_25_O_8_ 405.1549 (M+H^+^) found 405.1569.

### 3.2. Cell Culture

The human ovarian carcinoma cell line SKOV3 was obtained from the Korean Cell Line Bank (KCLB, Seoul, Korea) and maintained in RPMI 1640 supplemented with 10% fetal bovine serum (FBS) and 1× penicillin/streptomycin at 37 °C in 5% CO_2_ atmosphere. Cells were routinely checked for mycoplasma contamination and authenticated using STR DNA technology. These culture check was carried out three times.

### 3.3. Cell Viability

Cell viability was determined by the use of a CellTiter-Glo^®^ luminescent cell viability kit (Promega Corporation, Madison, WI, USA). After 4 × 10^3^ SKOV3 cells were seeded in an opaque-walled 96-well microplate, cells were treated with chemicals and incubated at 37 °C for 72 h. For equilibration of a microplate, it was incubated for 30 min at room temperature (RT). Thereafter, 100 µL of CellTiter-Glo^®^ reagent was added to 100 µL of medium containing cells and the plate was mixed for 2 min on an orbital shaker for inducing cell lysis. Finally, the plate was incubated for 10 min at RT to stabilize the luminescent signal and recorded by a GENios reader (Tecan, Männedorf, Switzerland). These tests were carried out three times.

## 4. Conclusions

The synthesis of a valuable C3-modified macrosphelide derivative was accomplished. The synthetic route includes a direct three-carbon introduction through propiolate addition and a sequential stereoselective reduction process. Employing this efficient synthetic route, biologically enhanced derivatives could be developed and utilized for macrosphelide-related anticancer drug discovery. Full disclosure of more advanced derivatives and the SAR studies of macrosphelides will be reported in due course.
